# Sex-specific differences in the relationship between fasting plasma glucose and carotid plaque in a cardiovascular high-risk population: a cross-sectional study

**DOI:** 10.3389/fendo.2025.1478640

**Published:** 2025-02-19

**Authors:** Mengjie Xu, Kai Xu, Weiguo Lin, Ruixue Sun, Shaorong Yan, Xiaoshu Chen, Yuzhan Lin

**Affiliations:** ^1^ Department of Laboratory Medicine, Wenzhou People’s Hospital, The Third Affiliated Hospital of Shanghai University, The Wenzhou Third Clinical College of Wenzhou Medical University, Wenzhou, China; ^2^ Department of Urology, The Third Affiliated Hospital of Wenzhou Medical University, Ruian, China; ^3^ Department of Clinical Laboratory, The Third Affiliated Hospital of Wenzhou Medical University, Ruian, China; ^4^ Department of Cardiology, Wenzhou People’s Hospital, The Third Affiliated Hospital of Shanghai University, The Wenzhou Third Clinical College of Wenzhou Medical University, Wenzhou, China

**Keywords:** fasting plasma glucose, carotid plaque, sex differences, cross sectional study, logistic regression

## Abstract

**Background:**

Despite extensive research on the epidemiological risk factors linking diabetes and carotid plaques, evidence regarding sex-specific differences remains scarce. This study aims to investigate the gender differences in the association between fasting blood glucose levels and the risk of carotid plaques among cardiovascular high-risk populations.

**Methods:**

This study used cross-sectional data from a large prospective cohort study. From 2019 to 2020, fasting blood glucose and carotid ultrasound examinations were conducted on high-risk cardiovascular populations in the pilot area. The independent variable was fasting blood glucose, and the dependent variable was the presence or absence of carotid plaques. A multivariable logistic regression model was used to calculate risk ratios. Generalized additive models combined with curve fitting were employed to assess the nonlinear relationship between fasting blood glucose levels and carotid plaques in the overall population and different genders.

**Results:**

This study included 1,063 cardiovascular high-risk patients. In the fully adjusted model, fasting blood glucose levels in men were positively associated with carotid plaques (OR = 1.21, 95% CI: 1.02-1.45, p = 0.0312). In women, fasting blood glucose levels were also positively associated with the risk of carotid plaque formation (OR = 1.15, 95% CI: 0.95-1.39). The generalized additive model results showed a linear relationship between fasting blood glucose levels and carotid plaques in the overall population. Stratified results indicated a linear relationship in men, while a nonlinear relationship was observed in women.

**Conclusion:**

There is an association between fasting blood glucose levels and carotid plaque, with an increased risk of carotid plaque as fasting blood glucose rises. Moreover, this relationship differs between sexes.

## Introduction

Carotid plaque formation is closely related to atherosclerosis. The growth of carotid plaques is often accompanied by plaque rupture (1, 2), playing a critical role in the development of adverse cardiovascular events such as ischemic stroke (3–5). Carotid ultrasound is commonly used as a screening tool for subclinical atherosclerosis, providing an assessment of cardiovascular disease risk independent of traditional risk factors (6, 7). However, the development of carotid plaques usually occurs without noticeable clinical symptoms for a long period before the onset of stroke.

The microvascular and macrovascular complications of diabetes are considered the origin of atherosclerosis (8). In people with diabetes, the risk of atherosclerotic cardiovascular disease increases by two to three times (9, 10). The main mechanism is the endothelial injury-inflammation response theory. High blood glucose induces oxidative stress and increases advanced glycation end products, causing arterial wall inflammation and endothelial dysfunction, leading to the accumulation of fatty streaks and fibrous plaques in the arteries (11, 12). From 2021 to 2045, the prevalence of diabetes is expected to increase from 10% to 12%, with the global affected population rising from 537 million to 783 million (9).

Blood glucose levels are a modifiable factor in the formation of carotid plaques. Additionally, early diagnosis of prediabetes and the establishment of healthy habits can help prevent the progression of diabetes and reduce cardiovascular risk. Currently, there is some controversy regarding the evidence on the relationship between blood glucose levels and carotid plaques (13–15). A study of 1,475 individuals found a significant association between glucose status and the occurrence of carotid plaques and stenosis (14). However, another meta-analysis found no significant association between impaired glucose tolerance and the incidence of carotid plaques in women (16). We hypothesized that the relationship between fasting blood glucose levels and carotid plaques differs between sexes. Therefore, this study utilized cross-sectional data from cardiovascular high-risk populations identified through community screening to investigate sex-specific differences in this association.

## Methods

### Study population

The cross-sectional study population was derived from high-risk cardiovascular individuals identified in the initial screening of the Zhejiang Province Wenzhou pilot of the large-scale prospective study (China PEACE Million Persons Project), established by the National Center for Cardiovascular Diseases of China in 2014 (17). The China PEACE Million Persons Project has been conducted continuously across China since 2014, and by 2023, the project has expanded to 383 pilots nationwide, screening millions of individuals (18). The inclusion criteria for the initial screening population in this study were individuals born between January 1, 1948, and December 31, 1988 (aged 35-75 years), who were permanent residents of the pilot project area, defined as having resided in the project area for at least six months in the 12 months prior to screening, and who voluntarily participated in the cardiovascular high-risk screening and signed an informed consent form.

### The initial screening process

During the initial screening process, project staff assessed cardiovascular disease risk by conducting preliminary inquiries about cardiovascular health, physical examinations, and rapid tests for blood glucose and lipids to identify high-risk individuals.

The diagnostic criteria for identifying high-risk cardiovascular patients are as follows: individuals meeting any one of the four criteria listed below are classified as high-risk.

1. History of myocardial infarction or stroke (ischemic or hemorrhagic), or undergoing Percutaneous Coronary Intervention (PCI) or Coronary Artery Bypass Grafting (CABG).
2. Systolic blood pressure (SBP) ≥160 mmHg or diastolic blood pressure (DBP) ≥100 mmHg.
3. Low density lipoprotein cholesterol (LDL-C) ≥160 mg/dL (4.14 mmol/L) or high density lipoprotein cholesterol (HDL-C) <30 mg/dL (0.78 mmol/L).
4. Cardiovascular risk was assessed based on the 2008 WHO guidelines for cardiovascular risk assessment and management (19), which utilize risk prediction charts considering factors such as age, sex, systolic blood pressure (measured twice and averaged, in mmHg), smoking status (current smokers or those who quit within the past year are considered smokers), diabetes status (previously diagnosed diabetes, use of hypoglycemic medications, or insulin injections), and total cholesterol (TC, mmol/L). If the ten-year cardiovascular disease risk is ≥20%, the individual is classified as high-risk.

### Questionnaire survey of cardiovascular high-risk population

Trained surveyors conducted baseline assessments using a structured questionnaire for all high-risk individuals, while physical examinations, laboratory tests, and ultrasound examinations were performed by experienced clinicians. The questionnaire included demographic variables (birthdate, sex, occupation, educational level, and marital status), family history of diseases (diabetes, cardiovascular diseases, cerebrovascular diseases, and dyslipidemia), self-reported cardiovascular risk factors (smoking habits, alcohol consumption, hypertension), and medication history. Additionally, menopausal status and age at menopause were assessed for female participants.

### Physical examination of high-risk patients

Physical examinations included measurements of height, weight, and waist circumference. Blood pressure measurements were taken using an electronic sphygmomanometer (HEM7211; Omron, Japan) calibrated by the metrology department. Subjects were seated for 5 minutes before measurement, avoiding any noticeable positional changes or emotional disturbances. Resting blood pressure was recorded on the right upper arm while the subject was seated. Blood pressure was measured twice with a 5-minute interval. If the difference between the two systolic measurements was greater than 10 mmHg, a third measurement was taken. The average of the two systolic (SBP) and diastolic (DBP) measurements was used.

### Laboratory examination

Fasting venous blood samples were collected using yellow-top vacuum blood collection tubes. Within 2 hours of collection, the whole blood samples were centrifuged at 1000-1200 g for 10-15 minutes at 4°C using a refrigerated centrifuge to separate the serum. All measurements were performed using the ROCHE c701 biochemical analyzer (ROCHE, Germany). Routine biochemical indicators included triglycerides (TG), total cholesterol (TC), fasting blood glucose, low-density lipoprotein cholesterol (LDL-C), and high-density lipoprotein cholesterol (HDL-C).

### Carotid ultrasound examination

Carotid arteries were assessed using a 7.5 MHz probe (Sonosite Micromaxx Ultrasound, Sonosite Inc, Bothell, WA, USA) for Doppler ultrasound. The patient was positioned supine with the neck rotated to the opposite side of the examination. Images were obtained from the distal wall of the common carotid artery, near the bifurcation, at three different angles, each covering a 1 cm segment.

### Variable definitions

Hypertension was defined as systolic blood pressure (SBP) ≥140 mmHg, diastolic blood pressure (DBP) ≥90 mmHg, self-reported history of hypertension, or current use of antihypertensive medications (20). Diabetes was defined as a fasting blood glucose level ≥7.0 mmol/L, any self-reported history of diabetes, or current use of diabetes medications (21). Alcohol consumption was classified as none, light drinking (1-2 drinks/day), moderate drinking (3-4 drinks/day), or heavy drinking (>5 drinks/day) according to definitions from the National Institute on Alcohol Abuse and Alcoholism (NIAAA) (22). Smoking status was classified based on survey information into three categories: non-smoker, former smoker, and current smoker (23). Female participants were categorized as postmenopausal or premenopausal based on the absence or presence of menstruation within the 12 months prior to recruitment (24, 25). Additionally, participants were further stratified into two groups based on the duration of menopause. We redefined patients’ education levels as binary variables: “high school or above” and “below high school”. Carotid plaque was diagnosed as local intimal thickening >1 mm or thickening >50% of the surrounding intima-media thickness (IMT).

Based on the WHO diabetes diagnostic criteria (26), we categorized the study participants into three groups: normal fasting glucose (NFG) for fasting blood glucose levels ≤6.0 mmol/L, impaired fasting glucose (IFG) for fasting blood glucose levels >6.0 and <7.0 mmol/L, and high fasting glucose (HFG) for fasting blood glucose levels ≥7.0 mmol/L.

### Statistical analysis

Continuous variables are expressed as means ± standard deviations or medians (interquartile ranges), while categorical variables are presented as frequencies or percentages. For continuous variables with a normal distribution, one-way ANOVA was used for between-group comparisons, while the Kruskal-Wallis test was used for non-normally distributed variables. Categorical variables were compared between groups using the chi-square test or Fisher’s exact test. Univariate logistic regression was used to examine the relationship between each variable and carotid plaque, while multivariate logistic regression models were used to calculate the odds ratio (OR) of fasting blood glucose levels for carotid plaque. Following the STROBE guidelines, we presented the results of the unadjusted, minimally adjusted, and fully adjusted multivariate regression models. Adjustment adequacy was determined based on whether the change in odds ratio was less than 10% when covariances were added to the model (27) and by the clinical interpretation between indicators. Trend tests were conducted for the mean fasting blood glucose levels in each group, treated as continuous variables.

The statistical analyses of this study were performed via R, version 4.2.0 (R Foundation) and EmpowerStats (http://www.empowerstats.com, X&Y Solutions, Inc., Boston, MA). The level of statistical significance was set at *P* < 0.05.

## Results

### Baseline characteristics of the subjects

**Figure 1:**
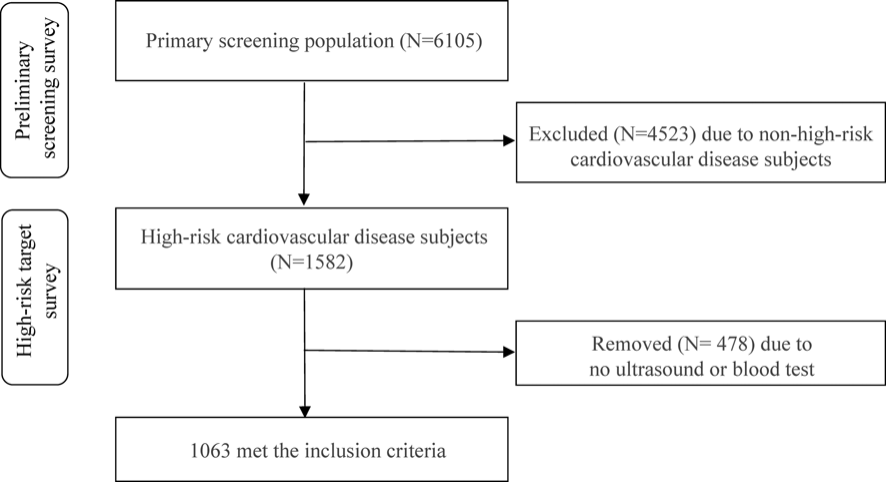
Flowchart of the subject selection.

A total of 6,105 residents participated in the initial screening, with 1,582 identified as high-risk cardiovascular patients. Among them, 478 lacked carotid ultrasound or blood test results, resulting in 1,063 patients included in the study (**Figure 1**). Of the total population, 445 were male (41.86%) and 618 were female (58.14%). Baseline clinical and biochemical characteristics of the participants are shown in **Table 1**. The NFG group consisted of 619 individuals (234 males and 385 females), the IFG group had 243 individuals (107 males and 136 females), and the HFG group included 201 individuals (104 males and 97 females). Among males, there were significant differences between fasting blood glucose level groups in terms of weight, BMI, waist circumference, ALT, AST, TG, smoking status, and use of lipid-lowering medications (P < 0.05). Among females, significant differences were observed between fasting blood glucose level groups in age, weight, BMI, waist circumference, ALT, prevalence of carotid plaques and hypertension, menopausal status and age at menopause, and use of lipid-lowering medications (P < 0.05).

**Table 1:** Baseline characteristics of participants (N =1063).

	Male				Female			
Variables	NFG	IFG	HFG	*P*	NFG	IFG	HFG	*P*
Number	234	107	104		385	136	97	
Demographic variables								
Age, years	62.97 ± 9.58	62.94 ± 8.97	62.05 ± 8.62	0.676	59.57 ± 8.43	62.72 ± 8.63	64.39 ± 7.38	<0.001*
Marital status, %				0.050				0.749
Others	0 (0.00)	3 (2.80)	2 (1.92)		17 (4.42)	7 (5.15)	3 (3.09)	
Married	234 (100.00)	104 (97.20)	102 (98.08)		368 (95.58)	129 (94.85)	94 (96.91)	
Education degree, %				0.700				0.337
Below high school	216 (92.31)	96 (89.72)	96 (92.31)		363 (94.29)	129 (94.85)	95 (97.94)	
Above high school	18 (7.69)	11 (10.28)	8 (7.69)		22 (5.71)	7 (5.15)	2 (2.06)	
Family income, %				0.492				0.700
<50000	58 (28.71)	24 (25.81)	30 (33.71)		129 (37.72)	42 (37.84)	35 (42.68)	
>50000	144 (71.29)	69 (74.19)	59 (66.29)		213 (62.28)	69 (62.16)	47 (57.32)	
Physical examinations								
Height, cm	167.36 ± 6.09	166.11 ± 6.02	167.06 ± 5.75	0.199	156.25 ± 5.38	156.03 ± 4.98	155.78 ± 5.61	0.722
Weight, kg	69.23 ± 9.72	69.75 ± 9.73	72.41 ± 10.26	0.022*	58.92 ± 8.07	60.90 ± 9.05	61.06 ± 8.21	0.013*
BMI, kg/m^2^	24.69 ± 3.00	25.22 ± 2.81	25.90 ± 3.14	0.003*	24.13 ± 3.05	24.99 ± 3.39	25.17 ± 3.20	0.002*
Waistline, cm	87.80 ± 7.66	88.26 ± 8.03	90.31 ± 8.60	0.027*	81.98 ± 8.45	84.72 ± 9.23	85.96 ± 8.16	<0.001*
SBP, mmHg	158.50 ± 19.68	159.95 ± 17.35	163.04 ± 16.36	0.113	152.37 ± 23.36	159.89 ± 20.09	162.91 ± 15.26	<0.001*
DBP, mmHg	89.09 ± 12.25	89.39 ± 10.76	90.22 ± 11.75	0.721	84.05 ± 12.39	84.92 ± 11.05	86.86 ± 10.24	0.108
Heart rate, bpm/min	71.72 ± 10.05	72.96 ± 10.71	74.68 ± 12.45	0.066	74.86 ± 9.30	76.40 ± 9.74	76.50 ± 9.50	0.131
Laboratory examination								
ALT, U/L	25.69 ± 11.75	31.11 ± 18.27	37.45 ± 38.66	<0.001*	21.68 ± 12.48	23.85 ± 14.37	27.05 ± 20.49	0.004*
AST, U/L	26.38 ± 8.10	28.38 ± 12.44	36.85 ± 66.14	0.026*	24.50 ± 8.78	24.68 ± 10.97	25.47 ± 16.08	0.728
AST/ALT	1.14 ± 0.41	1.01 ± 0.32	1.00 ± 0.37	0.001*	1.26 ± 0.45	1.15 ± 0.38	1.05 ± 0.35	<0.001*
Urea, mmol/L	5.89 ± 2.49	6.02 ± 1.85	5.57 ± 1.59	0.294	5.06 ± 1.51	5.48 ± 1.70	5.32 ± 1.81	0.022*
Creatinine, mmol/L	90.21 ± 62.14	84.49 ± 17.95	81.45 ± 18.13	0.240	64.19 ± 10.00	65.01 ± 12.35	63.55 ± 21.17	0.679
FBG, mmol/L	5.43 ± 0.35	6.37 ± 0.25	8.53 ± 1.36	<0.001*	5.43 ± 0.36	6.40 ± 0.28	8.31 ± 1.22	<0.001*
HDL-c, mmol/L	1.24 ± 0.33	1.22 ± 0.28	1.23 ± 0.33	0.840	1.35 ± 0.29	1.39 ± 0.32	1.35 ± 0.25	0.387
LDL-c, mmol/L	2.83 ± 0.81	2.83 ± 0.84	2.65 ± 0.98	0.162	3.07 ± 0.89	3.25 ± 0.90	3.03 ± 0.94	0.082
TC, mmol/L	5.03 ± 1.02	5.05 ± 1.06	5.10 ± 1.59	0.882	5.36 ± 1.08	5.55 ± 1.05	5.27 ± 1.17	0.113
TG, mmol/L	1.92 ± 1.47	2.02 ± 1.36	2.60 ± 2.87	0.007*	1.64 ± 1.18	1.55 ± 0.81	1.63 ± 0.85	0.675
Carotid plaque, %				0.928				0.002*
No	122 (52.14)	58 (54.21)	54 (51.92)		298 (77.40)	102 (75.00)	58 (59.79)	
Yes	112 (47.86)	49 (45.79)	50 (48.08)		87 (22.60)	34 (25.00)	39 (40.21)	
Cardiovascular risk factors								
Smoking, %				0.047*				0.320
Never	109 (46.58)	60 (56.07)	47 (45.19)		376 (97.66)	134 (98.53)	95 (97.94)	
Now	81 (34.62)	20 (18.69)	35 (33.65)		7 (1.82)	1 (0.74)	0 (0.00)	
Ever	44 (18.80)	27 (25.23)	22 (21.15)		2 (0.52)	1 (0.74)	2 (2.06)	
Alcohol, %				0.879				0.520
Never	108 (46.15)	42 (39.25)	45 (43.27)		283 (73.51)	97 (71.32)	78 (80.41)	
Light drinking	94 (40.17)	47 (43.93)	40 (38.46)		90 (23.38)	37 (27.21)	18 (18.56)	
Moderate drinking	19 (8.12)	11 (10.28)	11 (10.58)		9 (2.34)	2 (1.47)	1 (1.03)	
Excessive drinking	13 (5.56)	7 (6.54)	8 (7.69)		3 (0.78)	0 (0.00)	0 (0.00)	
Hypertension, %				0.103				<0.001*
No	25 (10.68)	8 (7.48)	4 (3.85)		86 (22.34)	18 (13.24)	5 (5.15)	
Yes	209 (89.32)	99 (92.52)	100 (96.15)		299 (77.66)	118 (86.76)	92 (94.85)	
Diabetes, %				<0.001*				<0.001*
No	208 (88.89)	76 (71.03)	0 (0.00)		367 (95.32)	100 (73.53)	0 (0.00)	
Yes	26 (11.11)	31 (28.97)	104 (100.00)		18 (4.68)	36 (26.47)	97 (100.00)	
Kidney disease, %				0.576				0.110
No	219 (99.55)	100 (100.00)	96 (98.97)		374 (98.94)	130 (97.74)	91 (95.79)	
Yes	1 (0.45)	0 (0.00)	1 (1.03)		4 (1.06)	3 (2.26)	4 (4.21)	
Menopausal status, %								<0.001*
No					111 (28.83%)	25 (18.38%)	8 (8.25%)	
Yes					274 (71.17%)	111 (81.62%)	89 (91.75%)	
Menopausal duration								0.048*
≤13years					118 (50.86%)	35 (36.08%)	34 (44.74%)	
>13years					114 (49.14%)	62 (63.92%)	42 (55.26%)	
Medication history								
Antihypertension medication, %				0.307				0.054
No	77 (36.84)	34 (34.34)	28 (28.00)		137 (45.82)	45 (38.14)	30 (32.61)	
Yes	132 (63.16)	65 (65.66)	72 (72.00)		162 (54.18)	73 (61.86)	62 (67.39)	
Antidiabetic medication, %				0.299				0.148
No	8 (30.77)	15 (48.39)	36 (34.62)		10 (55.56)	11 (30.56)	32 (32.99)	
Yes	18 (69.23)	16 (51.61)	68 (65.38)		8 (44.44)	25 (69.44)	65 (67.01)	
Antiplatelet medication, %				0.781				0.445
No	219 (93.59)	102 (95.33)	97 (93.27)		373 (96.88)	133 (97.79)	92 (94.85)	
Yes	15 (6.41)	5 (4.67)	7 (6.73)		12 (3.12)	3 (2.21)	5 (5.15)	
Antilipemic medication, %				0.009*				<0.001*
No	200 (85.47)	93 (86.92)	76 (73.08)		340 (88.31)	122 (89.71)	64 (65.98)	
Yes	34 (14.53)	14 (13.08)	28 (26.92)		45 (11.69)	14 (10.29)	33 (34.02)	

* *P*-value is significant.

Values are given as mean ± SD, medians with IQR or number (%).

NFG, normal fasting glucose; IFG, impaired fasting glucose; HFG, high fasting glucose; BMI, body mass index; DBP, diastole blood pressure; SBP, systolic blood pressure; HDL-c, high-density lipoprotein cholesterol; LDL-c, low-density lipoprotein cholesterol; AST, aspartate aminotransferase; ALT, alanine aminotransferase; FBG, blood glucose; TC, total cholesterol; TG, triglyceride.

### Results of the univariate analysis

The results of the univariate analysis are shown in **Table 2**. The univariate analysis revealed that in males, factors associated with carotid plaques included age, SBP, AST/ALT, hypertension, current smoking status, and use of antihypertensive medications. In female participants, significant associations with increased risk of carotid plaques were found with age, marital status, education degree, family income, SBP, urea, fasting blood glucose levels, triglycerides, hypertension, diabetes, menopausal status and age at menopause, and the use of antihypertensive, antidiabetic, lipid-lowering, and antiplatelet medications.

**Table 2:** Results of univariate analysis.

	Male		Female	
Variables	Statistics	OR (95%CI) *P*-value	Statistics	OR (95%CI) *P*-value
Demographic variables				
Age	62.89 ± 9.19	1.10 (1.07, 1.13) <0.001*	61.13 ± 8.49	1.10 (1.08, 1.13) <0.001*
Marital status				
Others	6 (1.30%)	Ref	29 (4.50%)	Ref
Married	454 (98.70%)	0.18 (0.02, 1.55) 0.119	615 (95.50%)	0.30 (0.14, 0.64) 0.002*
Education degree, %				
Below high school	421(91.52%)	Ref	613 (95.19%)	Ref
Above high school	39(8.48%)	0.35 (0.16, 073) 0.005*	31 (4.81%)	0.30 (0.09, 0.99) 0.049*
Family income, %				
<50000	115 (28.97%)	Ref	212 (38.20%)	Ref
>50000	282 (71.03%)	0.60 (0.38, 0.92) 0.020*	343 (61.80%)	0.56 (0.38, 0.83) 0.004*
Physical examinations				
Height	167.00 ± 6.03	0.99 (0.96, 1.02) 0.492	156.12 ± 5.29	0.97 (0.94, 1.00) 0.052
Weight	70.13 ± 9.98	0.98 (0.97, 1.00) 0.071	59.67 ± 8.34	0.98 (0.96, 1.00) 0.128
BMI	25.10 ± 3.03	0.94 (0.89, 1.00) 0.070	24.48 ± 3.16	0.98 (0.92, 1.04) 0.455
Waistline	88.51 ± 8.06	1.00 (0.98, 1.03) 0.814	83.28 ± 8.73	1.02 (1.00, 1.04) 0.123
SBP	159.95 ± 18.37	1.01 (1.00, 1.02) 0.007*	155.84 ± 22.00	1.02 (1.01, 1.03) <0.001*
DBP	89.42 ± 11.75	0.99 (0.98, 1.01) 0.487	84.55 ± 11.72	1.00 (0.98, 1.01) 0.668
Heart rate	72.90 ± 10.89	0.99 (0.98, 1.01) 0.334	75.42 ± 9.42	0.99 (0.97, 1.00) 0.143
Laboratory examination				
ALT	29.56 ± 22.59	1.00 (0.99, 1.01) 0.954	22.98 ± 14.44	0.99 (0.98, 1.00) 0.186
AST	29.17 ± 32.81	1.01 (0.99, 1.02) 0.245	24.76 ± 10.72	0.99 (0.97, 1.01) 0.162
AST/ALT	1.08 ± 0.38	1.80 (1.10, 2.96) 0.020*	1.20 ± 0.43	1.31 (0.87, 1.96) 0.191
Urea	5.89 ± 2.18	1.09 (0.99, 1.20) 0.065	5.19 ± 1.61	1.13 (1.01, 1.26) 0.027*
Creatinine	87.11 ± 46.45	1.00 (1.00, 1.00) 0.697	64.25 ± 12.92	1.01 (1.00, 1.03) 0.082
FBG	6.60 ± 1.99	1.08 (0.99, 1.19) 0.096	6.23 ± 1.58	1.18 (1.06, 1.31) 0.003*
HDL-c	1.23 ± 0.32	1.45 (0.81, 2.59) 0.210	1.36 ± 0.29	1.22 (0.67, 2.25) 0.513
LDL-c	2.80 ± 0.87	1.14 (0.92, 1.41) 0.216	3.11 ± 0.90	1.10 (0.90, 1.33) 0.355
TC	5.08 ± 1.20	1.03 (0.88, 1.20) 0.734	5.39 ± 1.09	1.16 (0.99, 1.37) 0.065
TG	2.11 ± 1.88	0.94 (0.85, 1.05) 0.282	1.62 ± 1.06	1.20 (1.03, 1.41) 0.022*
Cardiovascular risk factors				
Smoking				
Never	222 (48.26%)	Ref	631 (97.98%)	Ref
Now	140 (30.43%)	2.00 (1.30, 3.07) 0.002*	8 (1.24%)	2.97 (0.73, 12.01) 0.127
Ever	98 (21.30%)	1.25 (0.77, 2.02) 0.360	5 (0.78%)	1.98 (0.33, 11.95) 0.457
Alcohol				
Never	204 (44.35%)	Ref	474 (73.60%)	Ref
Light drinking	186 (40.43%)	0.90 (0.60, 1.34) 0.596	153 (23.76%)	1.24 (0.83, 1.86) 0.293
Moderate drinking	41 (8.91%)	0.95 (0.49, 1.86) 0.887	13 (2.02%)	0.25 (0.03, 1.93) 0.183
Excessive drinking	29 (6.30%)	0.53 (0.23, 1.19) 0.122	4 (0.62%)	0.00 (0.00, Inf) 0.975
Hypertension				
No	38 (8.26%)	Ref	113 (17.55%)	Ref
Yes	422 (91.74%)	3.22 (1.49, 6.97) 0.003*	531 (82.45%)	2.18 (1.26, 3.78) 0.005*
Diabetes				
No	287 (62.39%)	Ref	479 (74.38%)	Ref
Yes	173 (37.61%)	1.26 (0.86, 1.84) 0.228	165 (25.62%)	1.96 (1.33, 2.88) <0.001*
Menopausal status, %				
No			147 (22.83%)	Ref
Yes			497 (77.17%)	3.24(1.89,5.57) <0.0001*
Menopausal duration				
≤13years			194 (45.65%)	Ref
>13years			231 (54.35%)	2.56 (1.64, 4.01) <0.001*
Medication history				
Antihypertension medication				
No	146 (34.60%)	Ref	222 (41.81%)	Ref
Yes	276 (65.40%)	1.59 (1.06, 2.38) 0.025*	309 (58.19%)	2.05 (1.37, 3.08) <0.001*
Antidiabetic medication				
No	59 (34.10%)	Ref	59 (35.76%)	Ref
Yes	114 (65.90%)	1.15 (0.61, 2.16) 0.664	106 (64.24%)	2.08 (1.03, 4.20) 0.041*
Antiplatelet medication				
No	433 (94.13%)	Ref	623 (96.74%)	Ref
Yes	27 (5.87%)	0.87 (0.40, 1.89) 0.717	21 (3.26%)	6.25 (2.48, 15.78) <0.001*
Antilipemic medication				
No	382 (83.04%)	Ref	549 (85.25%)	Ref
Yes	78 (16.96%)	1.34 (0.82, 2.18) 0.244	95 (14.75%)	2.22 (1.40, 3.49) <0.001*
Kidney disease				
No	425 (99.30%)	Ref	618 (97.78%)	Ref
Yes	3 (0.70%)	2.17 (0.20, 24.05) 0.529	14 (2.22%)	1.16 (0.36, 3.77) 0.799

* *P*-value is significant.

Values are given as mean ± SD, medians with IQR or number (%).

CI, confidence interval; OR, odds ratio.

Inf: The model failed because of the small sample size.

### Relationship between fasting blood glucose levels and carotid plaque

We used multivariate logistic regression analysis to assess the association between fasting blood glucose levels and carotid plaques (**Table 3**). In the male population, the unadjusted model showed that fasting blood glucose levels were not significantly associated with the formation of carotid plaques (OR = 1.07, 95% CI: 0.94-1.22; P = 0.288). In the partially adjusted model (adjusting for age and BMI) and the fully adjusted model (adjusting for age, BMI, sex, marital status, education level, family income, smoking and drinking status, systolic and diastolic blood pressure, heart rate, ALT, AST, cholesterol, triglycerides, urea, and use of lipid-lowering medication), significant associations were observed: partially adjusted model (OR = 1.20, 95% CI: 1.03-1.40; P = 0.021) and fully adjusted model (OR = 1.21, 95% CI: 1.02-1.45; P = 0.031). In the female population, the unadjusted model showed a significant association between fasting blood glucose levels and arterial plaques (OR = 1.32, 95% CI: 1.15-1.53; p < 0.001). However, in the partially adjusted and fully adjusted models, the association between fasting blood glucose levels and plaque formation weakened, with ORs of 1.16 and 1.19, respectively. Among the different fasting blood glucose level groups, using the NFG group as a reference, the risk of carotid plaques was significantly higher in the HFG group (p-values < 0.05). In the HFG group, based on Model II, each one-unit increase in glucose was associated with an 88% higher risk of carotid plaques.

**Table 3:** Relationship between fasting blood glucose levels and carotid plaque in different genders.

	Non-adjusted model OR (95%CI) *P*	Mode I OR (95%CI) *P*	Model II OR (95%CI) *P*
Male			
Continuous variable			
Glucose	1.07 (0.94, 1.22) 0.288	1.20 (1.03, 1.40) 0.021*	1.21 (1.02, 1.45) 0.031*
Categorical variable			
NFG	Ref	Ref	Ref
IFG	0.92 (0.58, 1.46) 0.723	0.97 (0.56, 1.65) 0.901	1.09 (0.60, 1.96) 0.783
HFG	1.01 (0.64, 1.60) 0.971	1.59 (0.92, 2.77) 0.099	1.61 (0.87, 2.99) 0.131
P for trend	0.967	0.139	0.149
Female			
Continuous variable			
Glucose	1.32 (1.15, 1.53) <0.001*	1.16 (0.98, 1.38) 0.081	1.19 (0.99, 1.44)0.070
Categorical variable			
NFG	Ref	Ref	Ref
IFG	1.14 (0.72, 1.80) 0.569	0.90 (0.52, 1.54) 0.691	0.87 (0.49,1.55)0.634
HFG	2.30 (1.44, 3.69) <0.001*	1.74 (1.01, 3.01) 0.047*	1.88(1.04,3.42)0.038*
P for trend	0.001*	0.096	0.080

* *P*-value is significant.

Non-adjusted model adjusted for: None.

Model I adjust for: Age; BMI; Family income.

Model II model adjust for: Age; BMI; Family income; Education degree; Marital status; Smoking; Alcohol; SBP; DBP; Heart rate; LDL-C; Urea; AST; ALT; TC; TG; Antilipemic medication; menopausal status is adjusted for in the female population.

NFG, normal fasting glucose; IFG, impaired fasting glucose; HFG, high fasting glucose.

### Nonlinear relationship analysis

**Figure 2:**
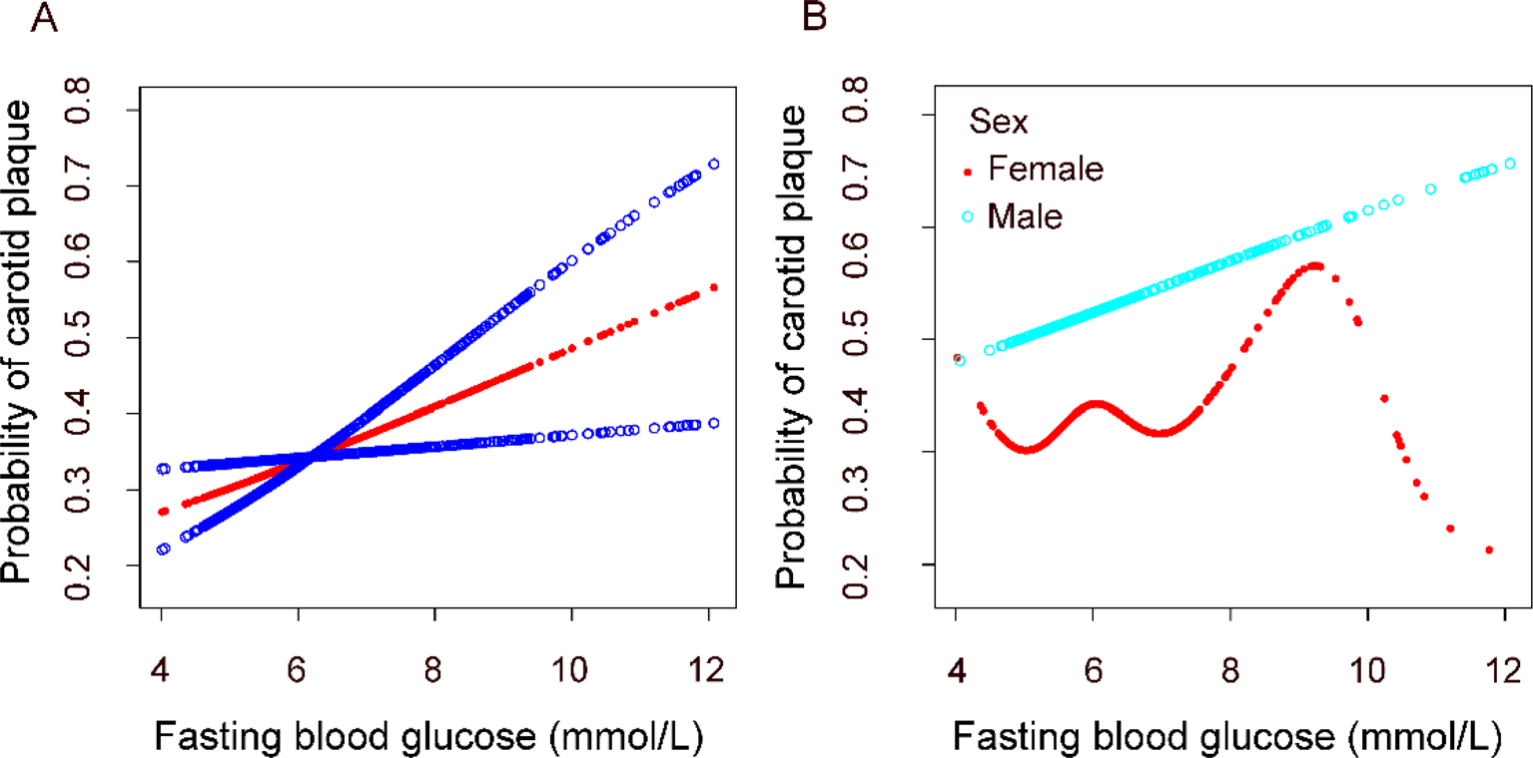
Association between fasting plasma glucose and carotid plaque. **(A)** in the general population. **(B)** in different gender groups. All analyses were adjusted for confounding factors including Age; BMI; Family income; Education degree; Marital status; Smoking; Alcohol; SBPUBP: Heart rate; LDL-C; Urea; Antilipemic medication.

We constructed fitting curves using a generalized additive model to analyze the nonlinear relationship between fasting blood glucose levels and carotid artery plaques in the overall population and across different genders (**Figure 2**). In the overall population, we found a linear relationship between fasting blood glucose levels and carotid artery plaques after adjusting for age, BMI, gender, marital status, education level, family income, smoking and alcohol consumption, systolic and diastolic blood pressure, heart rate, ALT, AST, cholesterol, triglycerides, urea, and lipid-lowering medication use. After stratifying by gender, we observed that the relationship between fasting blood glucose levels and carotid artery plaques remains linear in men but is nonlinear in women. Among females, stratified curve fitting was performed based on menopausal status and duration of menopause (**Supplementary Figure S1**). After adjusting for the same covariates, a differential association between glucose levels and carotid plaque risk was observed between postmenopausal and premenopausal women. Similarly, differences were also noted across groups with varying durations of menopause.

## Discussion

This cross-sectional survey of a community population revealed that higher fasting blood glucose levels were associated with an increased risk of carotid plaques in individuals at high cardiovascular risk. Further analysis indicated that the association between glucose levels and carotid plaque risk varied by sex. To our knowledge, this is the first study to report a sex-specific relationship between fasting glucose levels and carotid plaques. Moreover, our findings demonstrate that males are at a higher risk of carotid plaques compared to females. Postmenopausal women have a higher risk than premenopausal women, and women with longer menopausal durations exhibit a greater risk than those with shorter durations.

Numerous studies have investigated the relationship between fasting blood glucose levels and carotid artery plaques. Mostaza et al. (14) found that the prevalence of carotid atherosclerosis, assessed by the presence of carotid artery plaques, significantly increases as blood glucose status progresses from normal to prediabetes and diabetes among participants aged 45-74,but no sex differences were found according to diabetes diagnosis by fasting glucose. The study by Adeleke O. Fowokan (13) demonstrated that glucose could predict the size of carotid plaques and highlighted differences among ethnic groups. In contrast, our study revealed that the relationship between glucose levels and carotid plaques varies by sex. A cohort study conducted by Salvador Jr. et al. (28) identified a positive association between fasting blood glucose and the presence of carotid plaques, consistent with our findings. Our study further revealed sex-specific differences in the risk association between fasting blood glucose and carotid plaques. Some studies have reported contrary results. G. Brohall (16) conducted a meta-analysis of women aged 64 and found no significant association between impaired glucose tolerance and the incidence of carotid artery plaques. The author hypothesized that hormone replacement therapy (HRT) and cardiovascular medications might have influenced the results, though this hypothesis was not confirmed. In our study, cardiovascular-related medications were included as covariates in the analysis. We still observed an association between fasting blood glucose levels and carotid plaque risk in females, indicating that the relationship between fasting blood glucose and carotid plaques is independent of cardiovascular medication use.

The mechanisms underlying the relationship between blood glucose levels and carotid artery plaques remain unclear. Research has shown that high blood glucose mimics the effects of inflammatory mediators such as NF-kB, leading to endothelial cell activation and dysfunction, which are early stages in the development of atherosclerosis (11). Our study found that the relationship between fasting blood glucose levels and carotid artery plaques differs between men and women. A possible explanation is the presence of estrogen and its protective effect against coronary artery atherosclerosis (CAA). Studies have confirmed that estrogen significantly reduces the incidence of coronary artery atherosclerosis by improving vascular function and regulating lipid metabolism (29). The protective effects of estrogen on the vasculature may explain why women are less affected by high blood glucose-induced atherosclerosis. To test these hypotheses, we conducted stratified curve analyses based on menopausal status and duration of menopause in female participants. The results showed that postmenopausal women had a higher risk of carotid plaques compared to premenopausal women. Furthermore, women with longer menopausal durations were at greater risk than those with shorter durations.

This study has several limitations. First, as a cross-sectional study, we could only identify associations but could not establish causality. Additionally, participant selection was based on voluntary enrollment from residents at the project site. Although the refusal rate was low, selection bias cannot be completely ruled out. Moreover, as an observational study, the influence of numerous confounding factors cannot be excluded. To address this, we adjusted for various potential confounders in multivariable regression models. Menopausal status and duration, used as proxies for estrogen changes, were included in the analysis; however, these variables do not directly demonstrate the role of estrogen. Future research should further investigate the potential roles of factors such as inflammation and plasma corticosteroid levels in the development of carotid plaques. Our data are derived from middle-aged and elderly participants in a Chinese community, which may limit the generalizability of our findings to other populations.

## Conclusion

Our study demonstrates that fasting blood glucose levels are associated with the occurrence of carotid plaques in high-risk cardiovascular populations. Higher fasting blood glucose levels correlate with an increased risk of carotid plaques. Furthermore, this relationship differs between sexes.

## Data Availability

The raw data supporting the conclusions of this article will be made available by the authors, without undue reservation.
