# Supplementary figures and images for: Sex-specific differences in the relationship between fasting plasma glucose and carotid plaque in a cardiovascular high-risk population: a cross-sectional study
Source: Front Endocrinol (Lausanne). 2025 Feb 19;16:1478640. doi: 10.3389/fendo.2025.1478640 (PMC11880609; doi:10.3389/fendo.2025.1478640)

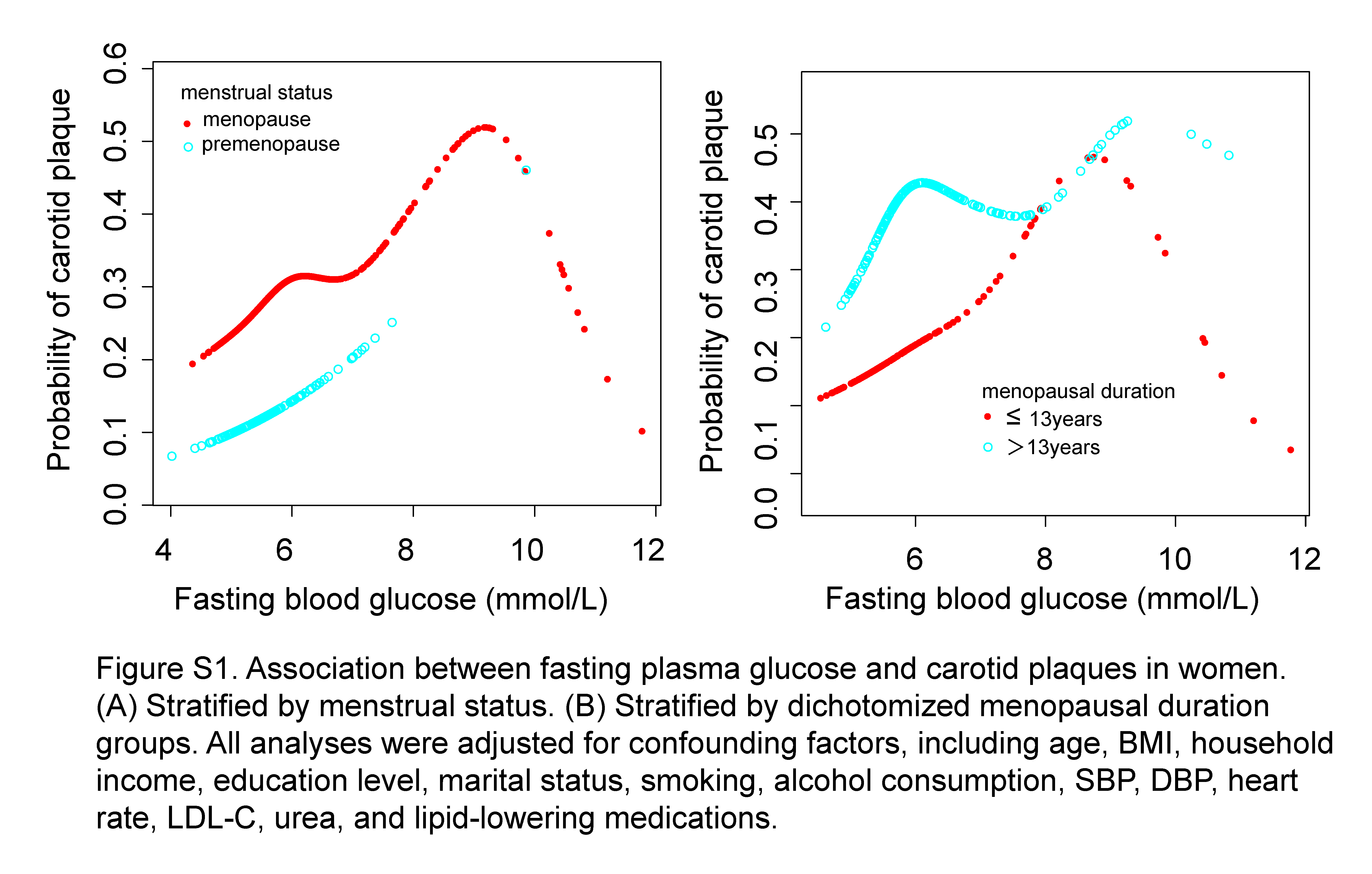

Supplement: Supplementary file 1 [file Image1.tif]
